# Spectroscopic Characterization of Bovine, Avian and Johnin Purified Protein Derivative (PPD) with High-Throughput Fourier Transform InfraRed-Based Method

**DOI:** 10.3390/pathogens8030136

**Published:** 2019-08-29

**Authors:** Sara Corneli, Laura Corte, Luca Roscini, Antonella Di Paolo, Claudia Colabella, Linda Petrucci, Giulio Severi, Monica Cagiola, Piera Mazzone

**Affiliations:** 1 Istituto Zooprofilattico Sperimentale dell’Umbria e delle Marche “Togo Rosati”, Via Gaetano Salvemini 1, 06126 Perugia, Italy; 2 Department of Pharmaceutical Sciences–Microbiology section, University of Perugia, Borgo 20 Giugno 74, 06121 Perugia, Italy

**Keywords:** purified protein derivative, mycobacteria, tuberculosis, FTIR, characterization

## Abstract

Tuberculins purified protein derivatives (PPDs) are obtained by precipitation from heat treated mycobacteria. PPDs are used in diagnosis of mycobacterial infections in humans and animals. Bovine PPD (PPDB) is obtained from *Mycobacterium bovis* (*Mycobacterium tuberculosis* complex), while Avian PPD (PPDA) and Johnin PPD (PPDJ) are extracted, respectively, from *Mycobacterium avium* and *M. avium subsp. paratuberculosis* (*M. avium* complex). PPDB and PPDA are used for bovine tuberculosis diagnosis, while PPDJ is experimentally used in the immunodiagnosis of paratuberculosis. Although PPDs date back to the 19th Century, limited knowledge about their composition is currently available. The goal of our study was to evaluate Fourier Transform InfraRed (FTIR) spectroscopy as a tool to differentiate PPDB, PPDA, and three PPDJs. The results highlighted that the three PPDs have specific profiles, correlated with phylogenetic characteristics of mycobacteria used for their production. This analysis is eligible as a specific tool for different PPDs batches characterization and for the assessment of their composition. The entire PPD production may be efficiently controlled, since the N content of each preparation is related to IR spectra, with a reference spectrum for each PPD and a standardized analysis protocol.

## 1. Introduction

Mycobacteria that belong to the *Mycobacterium* genus, family Mycobacteriaceae, are acid-fast bacteria that can affect human and animal populations. The genus *Mycobacterium* comprises more than 120 species [1], grouped mainly in *Mycobacterium tuberculosis* complex (MTBC), *Mycobacterium avium* complex (MAC), and *Mycobacterium* Other Than Tuberculosis (MOTT). Among the members of the MTBC, *Mycobacterium tuberculosis* and *Mycobacterium bovis* are very pathogenic mycobacteria. 

The first is primarily responsible for human tuberculosis (TB) [2] and the second one is the main causative agent of bovine tuberculosis (bTB) in domestic and wild animals [3]. MB can also be responsible for a zoonotic form of human TB, which cannot be differentiated at clinical examination, radiological and histopathological investigations from the TB due to *M. tuberculosis* [3,4].

The members of MAC are *Mycobacterium avium* subsp. *avium* (MA), *Mycobacterium avium* subsp. *paratuberculosis* (MAP), and others opportunistic pathogens are responsible for mycobacterial infections in animals and humans [5]. 

Traditionally, human TB diagnosis has been based on the use of tuberculin skin test (TST), which is also known as Mantoux Test. Nowadays, TST is still used in highly TB endemic area to diagnose latent *Mycobacterium tuberculosis* (LTBI) infection [6]. To date, indirect screening tests, as interferon gamma-release assays (IGRAs), are available to detect the presence of mycobacterial infections. In particular, the QuantiFERON®-TB Gold In-Tube test (QFT-GIT) and the T-SPOT-TB test (ELISPOT) can overtake some critical aspects of the TST, like the cross-reactivity in subjects that were vaccinated with Bacilli Calmette-Guerin (BCG) and measurement errors of the Skin reaction. For this reason, IGRAs should replace or support the TST [7].

The strength points of the bTB control are the efficient identification and prompt elimination of MB infected animals, which are responsible for the most of bTB outbreaks [8]. In many developed countries, bTB has been eliminated by eradication plans that are based on the use of TST, together with: i) mandatory culling of positive subjects, ii) movements limitation for infected herds, and iii) surveillance of slaughterhouses to reveal infected animal undetected *in vita* [8,9].

Tuberculin is the purified protein derivative (PPD) that was extracted from mycobacteria cultures in liquid synthetic medium, used routinely for TB diagnosis. In veterinary medicine, Bovine PPD (PPDB) is obtained from MB, while Avian PPD (PPDA) is extracted from MA. PPDs are employed in diagnostic tests that are provided by European Official Plans for the bovine tuberculosis eradication: PPDB is used for the single intradermal test (TST), while PPDA is used with PPDB for the comparative TST [10,11]. In order to increase the specificity of the TST, PPDA is added in the comparative TST to distinguish the MB infections from MA or MAP infections and to avoid cross-sensitization due to environmental mycobacteria. [12]. In veterinary medicine, as in human medicine, TST is based on a delayed-type hypersensitivity response (DTH) to intradermal injection of tuberculin [8] and on the subsequent swelling at the injection site in infected animals, measured 72 hours later [13].

Moreover, PPDB and PPDA are employed in the interferon-γ (IFN-γ) test, an ancillary test that quantifies the in vitro IFN-γ release in a whole blood culture under PPDs stimulation [14]. This assay, which is developed in veterinary medicine by Wood in the late 1980s, before the analogous QuantiFERON®-TB test, [15,16,17] is used to support TST. Identically to the QuantiFERON®-TB in human medicine, IFN-γ test detects cytokine produced by T lymphocytes of infected subjects, in response to stimulation with PPDs that are derived from tuberculous antigens [8].

Another mycobacteria disease affecting cattle is paratuberculosis (PTB) or Johne’s disease, a chronic inflammatory bowel disease of domestic ruminants and wildlife species worldwide [18,19,20]. MAP, the causal agent of PTB, has a zoonotic potential that has been questioned for a century since it was first claimed that Crohn’s disease in humans is pathologically and clinically comparable to PTB in animals [21]. Similar to classic tuberculin, Johnin (PPDJ) is a PPD that is obtained from a MAP culture in a liquid synthetic medium [13]. In cattle, the PPDJs can be used, only on an experimental basis, with the PPDB and PPDA, in lymphocyte stimulation of IFN-γ test, to verify their effectiveness in PTB diagnosis [22,23,24].

Although PPDs are immunological reagents that are widely used for diagnosis of bTB and studied by different authors, current knowledge regarding the exact composition of this heat-inactivated culture filtrate is lacking [25,26,27,28]. Since 1941, Seibert & Glen [29] declared that human PPD from *M. tuberculosis* consisted approximately in 92.9% protein, 5.9% polysaccharide, and 1.2% nucleic acid. The proteomic characterization of human and veterinary PPDs has been described by other authors [27,30], showing that PPD contains hundreds of different proteins. In fact, several studies defined PPDs as very heterogeneous compositions of proteins that range in size from very large aggregations to very small degraded segments [25,31,32,33]. In the study of Borsuk et al. [27], two bovine PPDs (Brazilian and English) and two avian PPDs (Brazilian and English) were characterized by LC-MS/MS to effectively study their protein profile; a total of 171 different proteins were identified, 77.9% of which was represented by cytoplasmic proteins, while 22.4% was represented by membrane or secreted proteins. Another type of approach provides a combination of proteomic and genomic studies: Santema et al. [34] studied and compared PPDB, PPDA, PPDJ from CVI-Lelystad (Lelystad, the Netherlands) and CZ Veterinaria (Pontevedra, Spain); Wynne et al. [26] characterized five different Australian PPDJs.

In our study, we performed a qualitative analysis of bovine PPD, avian PPD, and three experimental Johnin PPDs while using Fourier Transform InfraRed spectroscopy (FTIR). All of the PPDs were produced in Italy at Istituto Zooprofilattico Sperimentale of Umbria and Marche (IZSUM).

FTIR has been applied in microbiological studies for species identifications and strain characterizations [35,36,37,38,39,40,41,42,43,44,45,46,47]. FTIR-based bioassays are also useful for establishing the occurrence and entity of cellular stress, supposing that stressing conditions can change cellular metabolome before and after cell death [48,49]. FTIR analysis was used to provide the first in-depth studies on phenotype–genotype links in MB clones for the discrimination of MB strains [50] or specific procedure to classify mycobacteria at a species level, even for clinically isolated mycobacteria [51]. This method is able to identify the functional groups within both organic and inorganic molecules. It is possible to identify five regions in the spectrum: fatty acids, amides, mixed region, carbohydrates, and typing region [47]. All of the above-mentioned characteristics make the metabolomic FTIR fingerprint a valuable tool for the deep characterization of microbial cells.

This study represents the first application of FTIR analysis on purified protein derivative obtained from mycobacteria; FTIR analysis was used to characterize and differentiate the Italian PPDB, PPDA, and the three Johnin PPDs that are produced by IZSUM.

## 2. Results

### 2.1. Raw IR Spectra Analysis

**Figure 1:**
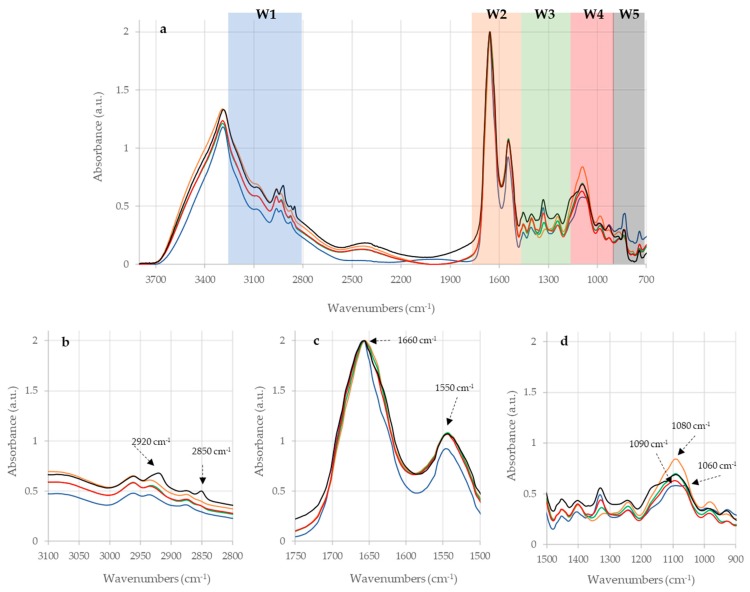
Overlaid InfraRed (IR) spectra of the five different purified protein derivatives (PPDs). **Legend.** Panel (**a**) average spectra of the five PPD analyzed; the different spectral regions are identified as follows: W1, containing fatty acids bands; W2 containing amides, proteins and peptides bands; W3, called Mixed region, containing bands from different types of molecules; W4, containing carbohydrates bands; W5, called fingerprinting region, containing unique absorbance peaks characteristic to specific microorganisms. Panel (**b**) enlargement of W1 region. Panel (**c**) enlargement of W2 region. Panel (**d**) enlargement of W4 region. Black line: PPD-A; blue line: PPD-B; orange line: PPD-JA; green line: PPD-JB; red line: PPD-JC. Major vibration peaks are labeled.

**Figure 2:**
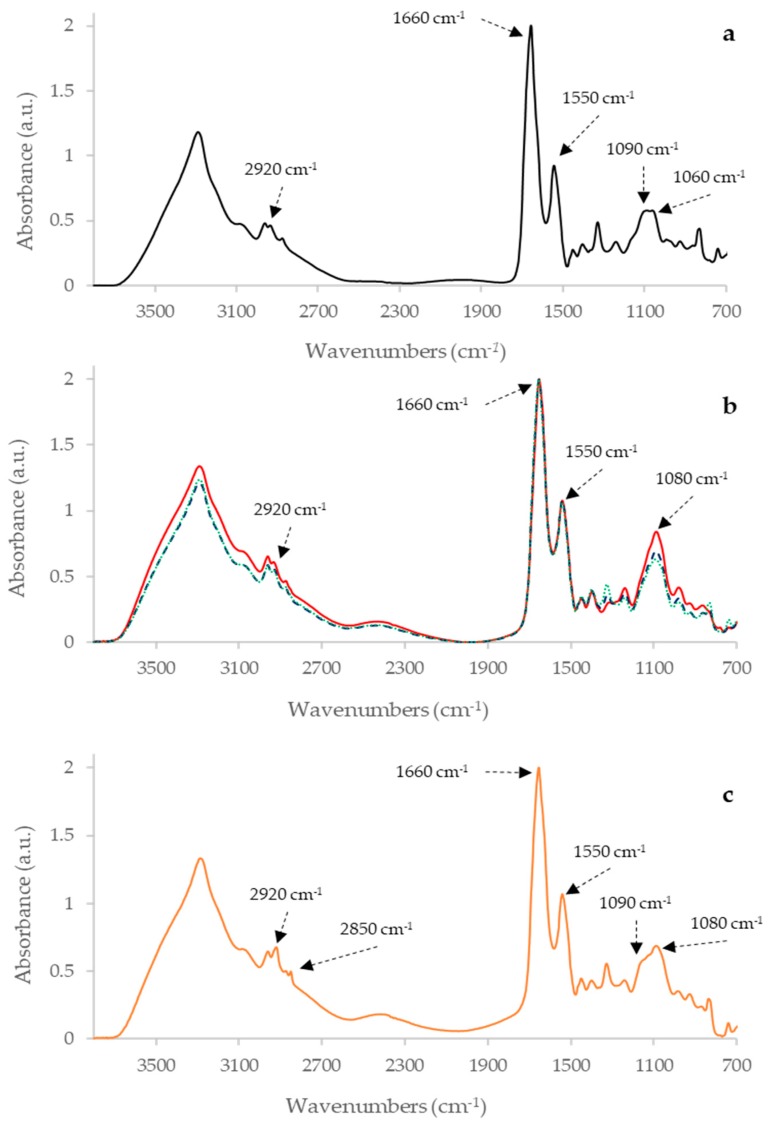
Separate Fourier Transform InfraRed (FTIR) spectra of Avian, Bovine and Johnin PPDs. Panel (**a**) Bovine PPD; Panel (**b**) Johnin PPDs: red solid line JA, blue dashed line JB and green dotted line JC PPD; Panel (**c**) Avian PPD. Major vibration peaks are labeled in each panel.

Four dilutions of each PPD were subjected to FTIR analysis, but only the undiluted samples produced significant spectra (reported in Supplementary Figure S2), so only these samples were considered for the subsequent analysis. The analysis of the IR spectra of the undiluted samples has shown that PPDB, PPDA, and the three PPDJ have different specific profile, as shown in Figure 1 and Figure 2.

This uniqueness is due to stretching and bending vibrations of bonds and the functional groups of each sample. The most prominent peaks are displayed in the amides and carbohydrates regions, which indicates that these are the most present components in the three PPDs (Bovine, Avian and Johnin PPD). The main bands are shown in Figure 1 and Figure 2, and Table 1 reports their assignments.

### 2.2. Statistical Analysis of IR Spectra

**Figure 3:**
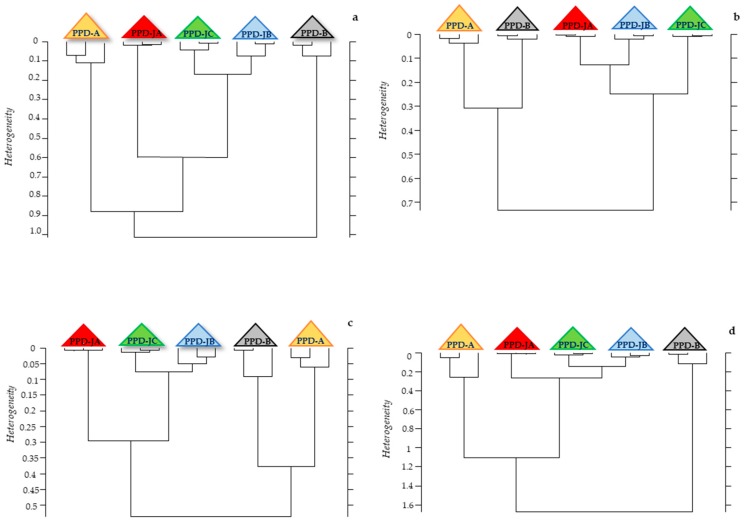
Hierarchical cluster analysis performed on the spectra of the five different PPDs. **Legend.** Panel (**a**) Clustering obtained analyzing IR vector normalized whole spectra; Panel (**b**) Clustering obtained analyzing W2 region; Panel (**c**) Clustering obtained analyzing W1 region; Panel (**d**) Clustering obtained analyzing W4 region.

Three different statistical analyses were performed on PPD spectra: hierarchical clustering, Partial Least Square (PLS) regression, and peak integration analysis. Hierarchical cluster analysis was performed while at first considering the whole spectrum and then each region taken individually. The dendrogram obtained considering the whole spectrum showed that the three PPDJs formed a clear group, while the other two, as obtained from MB and MA, are positioned on the sides (Figure 3a).

**Figure 4:**
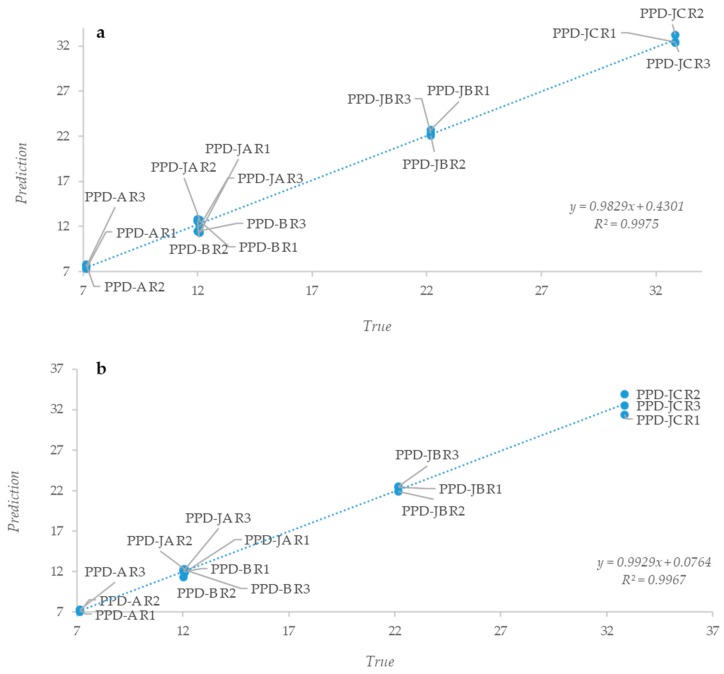
Partial Least Square (PLS) analysis showing the correlation between the spectra and the average nitrogen content of each PPD. **Legend.** Panel (**a**) Correlation between whole spectra and average nitrogen content; Panel (**b**) Correlation between amides region and the average nitrogen content. x-axis reports the real N content of each PPD, while y-axis reports the N content calculated by the regression model on the basis of the information contained in the spectra.

A similar discrimination was obtained while considering W1, W2, and W4 regions (Figure 3b–d), while it was not possible to obtain a clear separation of the different PPDs while considering the other regions (Supplementary Figure S1). The obtained IR spectra clearly indicated that the three PPDs types are different on the basis of their proteins’ spectral contribution. These results were expected, since the PPDs predominant protein component confers the immunogenic power and elicits a different immunological response in relation to the mycobacterium used in their production. Therefore, we carried out a PLS analysis to see whether it was possible to determine a linear predictive model that is useful to describe PPDs samples. Vector-normalized spectra were correlated with the average total nitrogen content of each PPD. For all of the analyzed samples, whole spectra showed a linear correlation with the nitrogen content (Figure 4a); similar results were only obtained when the analyzed spectral range was limited to the W2 region, which confirmed the importance of the protein component in the various PPDs (Figure 4b). 

Further proof of this prominent role of the protein component is given by integration analysis. The area under each peak was evaluated, obtaining an estimation of the total protein/nitrogen content for each analyzed sample, in relation to the area under the whole spectra. The ratio between the area under amides (W2) peak and the one under the whole spectrum (WS) was above 70% for almost all of the samples (Table 2). 

Therefore, it is evident that the protein component plays a key role in the PPDs differentiation; in fact, the proteins, which were obtained after TCA addition in the crude product (culture filtrate, culture secrete), are real antigens with immunogenic power, able to induce the cell-mediated response, detected in the animal with TST and IFN-γ test. Moreover, as regards the study of the three PPDJs, the use of MAP type strain ATCC 19698 in PPDJC allowed for performing a comparison with Johnins that were produced with Italian field strains, which showed an overlapping protein profile.

## 3. Discussion

PPDB and PPDA are commonly used in TST, as provided by European Community in the Official Plans for bTB eradication; they can be also used in IFN-γ test in support to TST, as a valuable tool in the ante mortem bTB diagnosis. Over the years, PPDJs have been used in the diagnosis of paratuberculosis, caused by MAP [23,24]. Therefore, several PPDJs in Europe, USA, and Australia were produced [26,52], but their efficiency to detect MAP infected animals in the IFN-γ test has not been defined, in part due to limited assay standardization and to the poor knowledge about these products.

In the last decade, many institutes have stopped PPDJs production and it has been difficult to find a European PPDJ. For this reason, in 2011, the IZSUM produced three batches of PPDJ, for experimental use in the IFN-γ test for the PTB diagnosis. The effectiveness of our bovine and avian PPDs is constantly checked in tests that were carried out on national territory, while the Johnin PPDs were evaluated within a research project funded by the Italian Ministry of Health (IZSUM 04/11 RC 2011). In the preliminary results of that research, 68 bovines from bTB Official Free herds were tested in the IFN-γ test with PPDB, PPDA, and three PPDJs; whole blood samples from each animal were stimulated with the five different tuberculins. PTB was diagnosed in 55 subjects with conventional tests provided by OIE Manual (IDVet ELISA, PCR, and bacterial cultures). In the IFN-γ test, as expected, the whole blood stimulated with PPDB gave negligible production of IFN-γ, while that stimulated with PPDA has produced a modest amount of IFN-γ. The blood that was stimulated with three PPDJs gave a significant IFN-γ production in 50 subjects PTB positive with a sensitivity of 78% [53]. The IFN-γ test highlights cell mediated response that occurs in animals that were infected with MAP or MB or MA. The blood samples in which lymphocytes produced IFN-γ after stimulation with PPDJs belong to animals that are infected with MAP. At the same time these subjects were not infected by MB, their lymphocytes stimulated with PPDB have not produced IFN-γ, since they had no immunological memory against MB. Finally, the production of IFN-γ by the lymphocytes of MAP infected animals, after stimulation with PPDA is due to the antigenic similarities between MAP and MA, since both belong to MAC. These results, which were obtained in PTB immunodiagnosis, reflect what occurs in in vivo tests for bTB diagnosis, TST, and IFN-γ test, in which the use of PPDB and PPDA is able to differentiate MB infections from those that are supported by mycobacteria belonging to the MAC. In fact, in terms of phylogeny, MB and MA belong to two different complexes, *M. tuberculosis* complex and *M. avium* complex, respectively [54,55,56]. A recent study [54], concerning the partial 16S rRNA sequences describes mycobacteria species and strains while using a neighbor joining phylogeny. The tree that was obtained using bootstrap supports based on 1,000 pseudo-replicates highlights the relationships between members of different *Mycobacterium* complex. MTBC members show more than 99.95% nucleotide sequence identity at the genome level [57], while the subspecies of the MAC share over 95% nucleotide sequence identity [54]. Data that were obtained through the FTIR analysis performed have confirmed what came out from phylogenetic characterization of mycobacteria used to produce PPDB, PPDA, and PPDJ, and they can support what occurs in vivo for the bTB and PTB immunodiagnosis.

Although PPDB and PPDA are very old tools, widely used for diagnosis of bTB, investigated in several studies, knowledge regarding those products is very limited [25,26,27,28]. Schiller et al. [58] adopted IFN-γ test in whole-blood cultures as a new tool to assess the diagnostic performance of the various PPDs. In this research, the results that are derived from the stimulation of the whole blood samples with different sources of tuberculins showed significant variability between PPDs from different manufacturers, and therefore they highlighted the importance of a better standardization of PPD products [25]. These studies demonstrate the importance of a constant control of PPDs for their biological potency differences that are hardly explainable for the complexity and variety of the PPDs molecular components [25].

In line with similar findings from other published data [59,60], the results of our analysis have demonstrated that PPDs are mainly constituted by a protein component, which, as reported [61,62,63], is able to stimulate cell-mediated immune response. Most PPDs proteins are extensively denatured by the preparation procedure (100 °C for three hours), and this can explain the problems in identifying and characterizing their constituents and their behavior in the activation of B and T cell [27]. Although most proteins of the PPDs are largely degraded, it has been demonstrated that, in those products, there are proteins that are resistant to heat degradation, such as MPB70 and MPB83 [61,62,63]. Protein degradation is not complete, since it leaves a number of intact peptide fragments that are useful for the identification with LC-MS/MS and probably responsible for the immunological properties of PPD. The detection of intact peptides in the PPDs is an important pathway to elucidate the immunologically active components of PPD [27].

In the study of Borsuk et al. [27], 21/171 proteins were identified in both bovine PPDs from Brazil and UK, but not in avian PPDs preparation; another group of 13 proteins was only present in this last PPD. Nine shared proteins were detected in all PPDs preparations. The proteome analysis that was carried out by Santema et al. [34], while using LC-MS/MS, showed that PPDs from the Netherlands and Spain share a high range of proteins with a high degree of homology, mainly between closely-related species as MAP and MA. Moreover, as reported by Roperto et al. [64], proteome analysis of the PPDB using bottom-up proteomics (protein digestion and nano-LC-MS/MS analysis) showed 198 proteins, which had not already been reported, and the proteomic pattern overlapping 80 proteins with the previous proteomes of the UK and Brazil PPDBs and 139 protein constituents of the Korean PPDBs. When PPDJ, PPDA, and PPDB protein component is compared, specific regions can be identified in these not degraded proteins, which provide pathogen specific immune responses against specific etiologic agents, such as MAP, MA, and MB.

FTIR spectra that were obtained from our samples have demonstrated that the three PPDs have specific profiles, which were correlated with phylogenetic characteristics of mycobacteria used for their production. Therefore, PPDJs are closely related to each other and they also show similarities with PPDA, as reported in the proteome analysis by Santema et al. [34], while Bovine PPD is set aside from the others.

FTIR spectroscopy has proven to be a fast, reliable, reproducible, and inexpensive technique, which is useful for describing and characterizing extracts, as well as substrates and cells [65,66,67].

This first application of FTIR analysis on PPDs showed that this method could be used to characterize and differentiate the PPDB, from the PPDA and from the Johnin PPD (Figure 1 and Figure 2). For the future it could be interesting to check whether the analysis is able to highlight differences between the batches of the same PPD preparations. It will be necessary to analyze several lots of the same PPD preparation in order to verify the capacity of the method to evaluate the reproducibility of the PPD production process and batches.

This preliminary study lay the foundations for a potential use of the FTIR analysis as a tool to characterize the different PPDs batches and to assess their composition; in fact, with a reference IR spectrum for each PPD and a standardized analysis protocol, the entire PPD production process might be controlled.

In fact, on the basis of existing correlation between the IR spectra and the average N content of each PPD, it could be possible to perform a further control on production protocols and on PPD batches. Moreover, mycobacteria cultures could be characterized with FTIR analysis before the heat inactivation in order to verify how the different production steps can affect PPD composition, so that their profile could be compared with those of the final products. Particularly, while associating FTIR with other analysis techniques, it could be interesting to check and identify the amount of proteins and peptides that are lost during the heat treatment of the mycobacterial culture filtrates, in order to increase the diagnostic power of the single tuberculins.

## 4. Materials and Methods 

### 4.1. PPDs Production

PPDs were extracted from cultures of mycobacteria in liquid synthetic medium. Bovine PPD was obtained from *M. bovis* AN5, Avium PPD from *M. avium* D4ER and the three Johnin PPDs from *M. avium* subsp. *paratuberculosis*. To obtain Johnin PPD, two MAP field strains and a MAP type strain were used. Twenty Italian MAP isolates were genotyped by amplification of Mini and Microsatellite loci [68]; strain A, widespread in Italian territory, and strain B, rare in Italy, were selected for the production respectively of PPDJA and PPDJB. MAP type strain ATCC 19,698 was used for the production of PPDJC. Briefly, mycobacteria cultures have been inactivated at 100 °C for three hours; cells were removed and the proteins extracted by precipitation with 40% trichloroacetic acid (TCA). Subsequently, material was washed, centrifuged, and resuspended several times with 1% TCA and physiological solution. Finally, the pellet was suspended in phosphate buffered saline and the total N determination was carried out by the Kjeldahl method [13,69].

### 4.2. FTIR-Based Characterization

PPDB, PPDA, and the three PPDJs were tested undiluted (10 µg/µL) and then added to test tubes containing HPLC-grade water in order to obtain ten-fold serial dilution (1 µg/µL; 0,1 µg/µL; 0,01 µg/µL). All of the tests were carried out in triplicate. 105 µL of each sample were used for three independent FTIR readings, 35 µL each according to the technique suggested by the work of Essendoubi et al. [70]. All the spectra were recorded in the range between 4000 and 400 cm^−1^. According to Helm and Naumann data [47,71], five major absorbance regions in the InfraRed (IR) spectra should be analyzed. Spectral regions (Windows, hereinafter referred as W) are defined, as follows: fatty acids (W1 or Region I) from 3000 to 2800 cm^−1^; amides (W2 or Region II) from 1700 to 1500 cm^−1^, containing the amide I and II bands of proteins and peptides; mixed region (W3 or Region III) from 1500 to 1200 cm^−1^, containing fatty acid-bending vibrations, proteins, and phosphate-carrying compounds; carbohydrates (W4 or Region IV) from 1200 to 900 cm^−1^; typing region (W5 or Region V) from 900 to 700 cm^−1^, called the “fingerprint region”, which contains weak but very unique absorbance peaks that are characteristic to specific microorganisms [72]. The FTIR experiments were carried out with a TENSOR 27 FTIR spectrometer, which was equipped with HTS-XT accessory for rapid automation of the analysis (BRUKER Optics GmbH, Ettlingen, Germany). FTIR measurements were performed in transmission mode. Spectral resolution was set at 4 cm^−1^, sampling 256 scans per sample. The software OPUS version 6.5 (BRUKER Optics GmbH, Ettlingen, Germany) was used to carry out the quality test, baseline correction and vector normalization.

### 4.3. Spectra Statistical Analyses

The FTIR data were subjected to hierarchical cluster analysis while using OPUS 6.5 software. The distance method selected was the Euclidean distance. The cluster analysis was performed considering first the whole spectrum and then the five different spectral regions already described previously in the methods section. The spectra were then subject to the QUANT2 algorithm, which is part of the OPUS 6.5 software (Bruker GmbH, Ettlingen, Germany). QUANT2 is a Partial Least Square (PLS) analysis and it makes a linear regression of the data generating two models: a first linear model, called *Prediction*, obtained from the regression based on the y values (intensities) given as initial data; and, a second model, called *True*, which is made up by x (wavenumbers) and y values given to the algorithm by the operator. The algorithm also calculates root mean square errors of cross validation (RMSECV). The recommended ranks exhibit the lowest RMSECV in the respective calibration methods. Typically, sufficiently low RMSECVs are reached with ranks higher than 3 indicating robust methods with low propensity to failure in predicting unknown samples. The predicted value is finally plotted versus the true value and the corresponding R^2^ value of the linear regression curves is reported (OPUS Manual, BRUKER Optics GmbH, Ettlingen, Germany).

### 4.4. Data Availability

Raw data will be made available to the readers upon request to the corresponding author.

## Figures and Tables

**Table 1:** Assignments of functional groups associated with major vibration bands highlighted in the spectra of the studied samples.

Sample	Wavenumbers (cm^−1^)	Definition of Band Assignments
**PPDA**	2920	Asymmetric stretching of CH_2_ in fatty acids ^1^
	2850	Symmetric stretching of CH_2_ in fatty acids ^1^
	1660	Amide I of α-helical structure ^1^
	1550	Amide II ^1^
	1160	stretching of CC and bending of COP and COH in DNA and RNA backbone ^1^
	1080	DNA and RNA backbones C-C symmetric stretching ^1^
**PPDB**	2920	Asymmetric stretching of CH_2_ in fatty acids ^1^
	1660	α-helix of Amide I ^1^
	1550	Amide II ^1^
	1090	Symmetric stretching of P = O of nucleic acids and phospholipids ^1^
	1060	P=O absorption band of nucleic acids and phospholipids ^2^
**PPDJ (A, B and C)**	2920	Asymmetric stretching of CH_2_ in fatty acids ^1^
	1660	α-helix of Amide I ^1^
	1550	Amide II ^1^
	1080	DNA and RNA backbones C-C symmetric stretching ^1^

^1^—Yu C. and Irudayaraj J., *Biopolymers*, 2005, **77.6**, 368–377. ^2^—Hancock A.J. and Kates M., *Journal of lipid research*, 1973, **14.4**, 422–429.

**Table 2:** Average integration area under the spectra of the studied PPDs.

	*PPD-A*	*PPD-B*	*PPD-JA*	*PPD-JB*	*PPD-JC*
***Average W1 Peak***	14.859	11.844	12.096	13.187	12.421
***Average W2 Peak***	175.482	183.900	189.907	191.089	189.649
***Average W3 Peak***	6.329	3.339	13.418	11.288	8.394
***Average W4 Peak***	36.162	35.787	59.996	50.583	45.902
***Average W5 Peak***	0.621	0.556	0.359	0.303	0.435
***Average WS Sum***	233.453	235.426	275.775	266.449	256.801
***Average W1/WS***	0.064	0.050	0.044	0.049	0.048
***Average W2/WS***	**0.752**	**0.781**	**0.689**	**0.717**	**0.739**
***Average W3/WS***	0.027	0.014	0.049	0.042	0.033
***Average W4/WS***	0.155	0.152	0.218	0.190	0.179
***Average W5/WS***	0.003	0.002	0.001	0.001	0.002

## Supplementary Materials

The following are available online at https://www.mdpi.com/2076-0817/8/3/136/s1, Supplementary Figure S1. Hierarchical cluster analysis performed on the spectra of the five different PPDs. **Legend.** Panel a. Clustering obtained analyzing W3 region; Panel b. Clustering obtained analyzing W5 region. Supplementary Figure S2. Raw FTIR spectra of Avian, Bovine and Johnin PPDs. **Legend**. Each sample is presented in triplicate. The five PPDs are represented by the different colors as detailed in the following lines: Blue = Avian PPD; Red = Bovine PPD; Green = Johnin PPD A; Orange = Johnin PPD B; Black = Johnin PPD C.

## References

[1] Tortoli E. (2006). The new mycobacteria: An update. FEMS Immunol. Med. Microbiol..

[2] Romha G., Gebru G., Asefa A., Mamo G. (2018). Epidemiology of *Mycobacterium bovis* and *Mycobacterium tuberculosis* in animals: Transmission dynamics and control challenges of zoonotic TB in Ethiopia. Prev. Vet. Med..

[3] De la Rua-Domenech R. (2006). Human *Mycobacterium bovis* infection in the United Kingdom: Incidence, risks, control measures and review of the zoonotic aspects of bovine tuberculosis. Tuberculosis.

[4] Kubica T., Rusch-Gerdes S., Niemann S. (2003). *Mycobacterium bovis* subsp. *caprae* caused one-third of human M. bovis-associated tuberculosis cases reported in Germany between 1999 and 2001. J. Clin. Microbiol..

[5] Moravkova M., Hlozek P., Beran V., Pavlik I., Preziuso S., Cuteri V., Bartos M. (2008). Strategy for the detection and differentiation of *Mycobacterium avium* species in isolates and heavily infected tissues. Res. Vet. Sci..

[6] World Health Organization (2018). Latent Tuberculosis Infection: Updated and Consolidated Guidelines for Programmatic Management.

[7] Auguste P., Tsertsvadze A., Pink J., Court R., McCarthy N., Sutcliffe P., Clarke A. (2017). Comparing interferon-gamma release assays with tuberculin skin test for identifying latent tuberculosis infection that progresses to active tuberculosis: Systematic review and meta-analysis. BMC Infect. Dis..

[8] De la Rua-Domenech R., Goodchild A., Vordermeier H., Hewinson R., Christiansen K., Clifton-Hadley R. (2006). Ante mortem diagnosis of tuberculosis in cattle: A review of the tuberculin tests, γ-interferon assay and other ancillary diagnostic techniques. Res. Vet. Sci..

[9] Chen Y., Danelishvili L., Rose S.J., Bermudez L.E. (2019). *Mycobacterium bovis* BCG Surface Antigens Expressed under the Granuloma-Like Conditions as Potential Inducers of the Protective Immunity. Int J. Microbiol..

[10] European Community (2002). Commission Regulation (EC) 1226/2002 of 8 July 2002 amending Annex B to Council Directive 64/432/EEC. Off. J. Eur. Commun..

[11] EU Council (1964). EU Council Directive 64/432/EEC. https//eur-lex.europa.eu/eli/dir/1964/432/oj/.

[12] Clegg T.A., Good M., Doyle M., Duignan A., More S.J., Gormley E. (2017). The performance of the interferon gamma assay when used as a diagnostic or quality assurance test in *Mycobacterium bovis* infected herds. Prev. Vet. Med..

[13] World Organisation for Animal Health (OIE) (2009). Manual of Diagnostic Tests and Vaccines for Terrestrial Animals.

[14] Eirin M.E., Macias A., Magnano G., Morsella C., Mendez L., Blanco F.C., Bianco M.V., Severina W., Alito A., de los Angeles Pando M. (2015). Identification and evaluation of new *Mycobacterium bovis* antigens in the in vitro interferon gamma release assay for bovine tuberculosis diagnosis. Tuberculosis Edinb..

[15] Wood P.R., Jones S.L. (2001). BOVIGAM: An in vitro cellular diagnostic test for bovine tuberculosis. Tuberculosis Edinb..

[16] Wood P.R., Rothel J.S. (1994). In vitro immunodiagnostic assays for bovine tuberculosis. Vet. Microbiol..

[17] Wood P.R., Corner L.A., Rothel J.S., Baldock C., Jones S.L., Cousins D.B., McCormick B.S., Francis B.R., Creeper J., Tweddle N.E. (1991). Field comparison of the interferon-gamma assay and the intradermal tuberculin test for the diagnosis of bovine tuberculosis. Aust. Vet. J..

[18] Chiodini R.J., Chamberlin W.M., Sarosiek J., McCallum R.W. (2012). Crohn’s disease and the mycobacterioses: A quarter century later. Causation or simple association?. Crit. Rev. Microbiol..

[19] Harris N.B., Barletta R.G. (2001). *Mycobacterium avium* subsp. *paratuberculosis* in Veterinary Medicine. Clin. Microbiol. Rev..

[20] Chiodini R.J., Van Kruiningen H.J., Merkal R.S. (1984). Ruminant paratuberculosis (Johne’s disease): The current status and future prospects. Cornell Vet..

[21] Waddell L.A., Rajic A., Stark K.D., McEwen S.A. (2015). The zoonotic potential of *Mycobacterium avium* ssp. *paratuberculosis*: A systematic review and meta-analyses of the evidence. Epidemiol. Infect..

[22] Jungersen G., Mikkelsen H., Grell S.N. (2012). Use of the johnin PPD interferon-gamma assay in control of bovine paratuberculosis. Vet. Immunol. Immunopathol..

[23] Kalis C., Collins M., Hesselink J., Barkema H. (2003). Specificity of two tests for the early diagnosis of bovine paratuberculosis based on cell-mediated immunity: The Johnin skin test and the gamma interferon assay. Vet. Microbiol..

[24] Jungersen G., Huda A., Hansen J.J., Lind P. (2002). Interpretation of the gamma interferon test for diagnosis of subclinical paratuberculosis in cattle. Clin. Diagn. Lab. Immunol..

[25] Yang H., Kruh-Garcia N.A., Dobos K.M. (2012). Purified protein derivatives of tuberculin--past, present, and future. FEMS Immunol. Med. Microbiol..

[26] Wynne J.W., Shiell B.J., Colgrave M.L., Vaughan J.A., Beddome G., Michalski W.P. (2012). Production and proteomic characterisation of purified protein derivative from *Mycobacterium avium* subsp. *paratuberculosis*. Proteome Sci..

[27] Borsuk S., Newcombe J., Mendum T.A., Dellagostin O.A., McFadden J. (2009). Identification of proteins from tuberculin purified protein derivative (PPD) by LC-MS/MS. Tuberculosis Edinb..

[28] Kuwabara S., Tsumita T. (1974). Letter: Primary structure of tuberculin-active protein from tubercule bacilli. Jpn. J. Exp. Med..

[29] Seibert F., Glen J. (1941). PPD-S was comprised of approximately 92.1% protein, 5.9% polysaccharides and 1.2% nucleic acid. Am. Rev. Tuberc..

[30] Cho Y.S., Dobos K.M., Prenni J., Yang H., Hess A., Rosenkrands I., Andersen P., Ryoo S.W., Bai G.H., Brennan M.J. (2012). Deciphering the proteome of the in vivo diagnostic reagent “purified protein derivative” from *Mycobacterium tuberculosis*. Proteomics.

[31] Ho M.M., Kairo S.K., Corbel M.J. (2006). Tuberculin purified protein derivative (PPD) immunoassay as an in vitro alternative assay for identity and confirmation of potency. Hum. Vaccin..

[32] Rowland S.S., Ruckert J.L., Cummings P.J. (1999). Low molecular mass protein patterns in mycobacterial culture filtrates and purified protein derivatives. FEMS Immunol. Med. Microbiol..

[33] Klausen J., Magnusson M., Andersen A.B., Koch C. (1994). Characterization of purified protein derivative of tuberculin by use of monoclonal antibodies: Isolation of a delayed-type hypersensitivity reactive component from *M. tuberculosis* culture filtrate. Scand. J. Immunol..

[34] Santema W., Overdijk M., Barends J., Krijgsveld J., Rutten V., Koets A. (2009). Searching for proteins of *Mycobacterium avium* subspecies *paratuberculosis* with diagnostic potential by comparative qualitative proteomic analysis of mycobacterial tuberculins. Vet. Microbiol..

[35] Roscini L., Corte L., Antonielli L., Rellini P., Fatichenti F., Cardinali G. (2010). Influence of cell geometry and number of replicas in the reproducibility of whole cell FTIR analysis. Analyst.

[36] Del Bove M., Lattanzi M., Rellini P., Pelliccia C., Fatichenti F., Cardinali G. (2009). Comparison of molecular and metabolomic methods as characterization tools of *Debaryomyces hansenii* cheese isolates. Food Microbiol..

[37] Szeghalmi A., Kaminskyj S., Gough K.M. (2007). A synchrotron FTIR microspectroscopy investigation of fungal hyphae grown under optimal and stressed conditions. Anal. Bioanal. Chem..

[38] Fischer G., Braun S., Thissen R., Dott W. (2006). FT-IR spectroscopy as a tool for rapid identification and intra-species characterization of airborne filamentous fungi. J. Microbiol. Methods.

[39] Adt I., Toubas D., Pinon J.M., Manfait M., Sockalingum G.D. (2006). FTIR spectroscopy as a potential tool to analyse structural modifications during morphogenesis of *Candida albicans*. Arch. Microbiol..

[40] Yu C., Irudayaraj J. (2005). Spectroscopic characterization of microorganisms by Fourier transform infrared microspectroscopy. Biopolymers.

[41] Zhao H., Kassama Y., Young M., Kell D.B., Goodacre R. (2004). Differentiation of *Micromonospora* isolates from a coastal sediment in Wales on the basis of Fourier transform infrared spectroscopy, 16S rRNA sequence analysis, and the amplified fragment length polymorphism technique. Appl. Environ. Microbiol..

[42] Lai S., Goodacre R., Manchester L.N. (2004). Whole-organism fingerprinting of the genus *Carnobacterium* using Fourier transform infrared spectroscopy (FT-IR). Syst. Appl. Microbiol..

[43] Wenning M., Seiler H., Scherer S. (2002). Fourier-transform infrared microspectroscopy, a novel and rapid tool for identification of yeasts. Appl. Environ. Microbiol..

[44] Oberreuter H., Seiler H., Scherer S. (2002). Identification of coryneform bacteria and related taxa by Fourier-transform infrared (FT-IR) spectroscopy. Int. J. Syst. Evol. Microbiol..

[45] Kummerle M., Scherer S., Seiler H. (1998). Rapid and reliable identification of food-borne yeasts by Fourier-transform infrared spectroscopy. Appl. Environ. Microbiol..

[46] Goodacre R., Timmins E.M., Rooney P.J., Rowland J.J., Kell D.B. (1996). Rapid identification of *Streptococcus* and *Enterococcus* species using diffuse reflectance-absorbance Fourier transform infrared spectroscopy and artificial neural networks. FEMS Microbiol. Lett..

[47] Helm D., Labischinski H., Schallehn G., Naumann D. (1991). Classification and identification of bacteria by Fourier-transform infrared spectroscopy. J. Gen. Microbiol..

[48] Corte L., Antonielli L., Roscini L., Fatichenti F., Cardinali G. (2011). Influence of cell parameters in Fourier transform infrared spectroscopy analysis of whole yeast cells. Analyst.

[49] Corte L., Rellini P., Roscini L., Fatichenti F., Cardinali G. (2010). Development of a novel, FTIR (Fourier transform infrared spectroscopy) based, yeast bioassay for toxicity testing and stress response study. Anal. Chim. Acta.

[50] Winder C.L., Gordon S.V., Dale J., Hewinson R.G., Goodacre R. (2006). Metabolic fingerprints of *Mycobacterium bovis* cluster with molecular type: Implications for genotype-phenotype links. Microbiology.

[51] Rebuffo-Scheer C.A., Kirschner C., Staemmler M., Naumann D. (2007). Rapid species and strain differentiation of non-tubercoulous mycobacteria by Fourier-Transform Infrared microspectroscopy. J. Microbiol. Methods.

[52] Semret M., Bakker D., Smart N., Olsen I., Haslov K., Behr M.A. (2006). Genetic analysis of *Mycobacterium avium* complex strains used for producing purified protein derivatives. Clin. Vacc. Immunol. CVI.

[53] Mazzone P., Corneli S., Vitale N., Di Paolo A., Biagetti M., Maresca C., Ricchi M., Mangili P., Papa P., Pezzotti G. Use of new johnin in gamma-IFN test to detect *Mycobacterium avium* subsp. *paratuberculosis* (MAP) infected cattle: Preliminary data. Proceedings of the XII International Colloquium on Paratuberculosis.

[54] Rue-Albrecht K., Magee D.A., Killick K.E., Nalpas N.C., Gordon S.V., MacHugh D.E. (2014). Comparative functional genomics and the bovine macrophage response to strains of the mycobacterium genus. Front. Immunol..

[55] Dvorska L., Bartos M., Martin G., Erler W., Pavlik I. (2001). Strategies for differentiation, identification and typing of medically important species of mycobacteria by molecular methods. Vet. Med. PRAHA.

[56] Rogall T., Wolters J., Flohr T., Bottger E.C. (1990). Towards a phylogeny and definition of species at the molecular level within the genus *Mycobacterium*. Int. J. Syst. Bacteriol..

[57] Brosch R., Gordon S.V., Marmiesse M., Brodin P., Buchrieser C., Eiglmeier K., Garnier T., Gutierrez C., Hewinson G., Kremer K. (2002). A new evolutionary scenario for the *Mycobacterium tuberculosis* complex. Proc. Natl. Acad. Sci. USA.

[58] Schiller I., Vordermeier H.M., Waters W.R., Kyburz A., Cagiola M., Whelan A., Palmer M.V., Thacker T.C., Meijlis J., Carter C. (2010). Comparison of tuberculin activity using the interferon-gamma assay for the diagnosis of bovine tuberculosis. Vet. Rec..

[59] Capsel R.T., Thoen C.O., Reinhardt T.A., Lippolis J.D., Olsen R., Stabel J.R., Bannantine J.P. (2016). Composition and Potency Characterization of *Mycobacterium avium* subsp. *paratuberculosis* Purified Protein Derivatives. PLoS ONE.

[60] Prasad T.S., Verma R., Kumar S., Nirujogi R.S., Sathe G.J., Madugundu A.K., Sharma J., Puttamallesh V.N., Ganjiwale A., Myneedu V.P. (2013). Proteomic analysis of purified protein derivative of *Mycobacterium tuberculosis*. Clin. Proteomics.

[61] Wiker H.G. (2004). Unmatched sequences in public databases—Exemplified by tuberculin-active protein. Scand. J. Immunol..

[62] Harboe M., Nagai S., Wiker H.G., Sletten K., Haga S. (1995). Homology between the MPB70 and MPB83 proteins of *Mycobacterium bovis* BCG. Scand. J. Immunol..

[63] Harboe M., Wiker H.G., Lachmann P.J. (1990). Carrier effect of concanavalin A-reactive and -non-reactive material in tuberculin PPD. Scand. J. Immunol..

[64] Roperto S., Varano M., Russo V., Luca R., Cagiola M., Gaspari M., Ceccarelli D.M., Cuda G., Roperto F. (2017). Proteomic analysis of protein purified derivative of *Mycobacterium bovis*. J. Transl. Med..

[65] Colabella C., Corte L., Roscini L., Shapaval V., Kohler A., Tafintseva V., Tascini C., Cardinali G. (2017). Merging FT-IR and NGS for simultaneous phenotypic and genotypic identification of pathogenic *Candida* species. PLoS ONE.

[66] Corte L., Dell’abate M.T., Magini A., Migliore M., Felici B., Roscini L., Sardella R., Tancini B., Emiliani C., Cardinali G. (2014). Assessment of safety and efficiency of nitrogen organic fertilizers from animal-based protein hydrolysates--a laboratory multidisciplinary approach. J. Sci. Food Agric..

[67] Corte L., di Cagno R., Groenewald M., Roscini L., Colabella C., Gobbetti M., Cardinali G. (2015). Phenotypic and molecular diversity of *Meyerozyma guilliermondii* strains isolated from food and other environmental niches, hints for an incipient speciation. Food Microbiol..

[68] Ricchi M., Barbieri G., Taddei R., Belletti G.L., Carra E., Cammi G., Garbarino C.A., Arrigoni N. (2011). Effectiveness of combination of Mini-and Microsatellite loci to sub-type *Mycobacterium avium* subsp. *paratuberculosis* Italian type C isolates. BMC Vet. Res..

[69] Helrich K. (1990). Official Methods of Analysis of the AOAC.

[70] Essendoubi M., Toubas D., Bouzaggou M., Pinon J.M., Manfait M., Sockalingum G.D. (2005). Rapid identification of *Candida* species by FT-IR microspectroscopy. Biochim. Biophys. Acta.

[71] Naumann D., Helm D., Labischinski H. (1991). Microbiological characterizations by FT-IR spectroscopy. Nature.

[72] Tang M., McEwen G.D., Wu Y., Miller C.D., Zhou A. (2013). Characterization and analysis of mycobacteria and Gram-negative bacteria and co-culture mixtures by Raman microspectroscopy, FTIR, and atomic force microscopy. Anal. Bioanal. Chem..

