# Supplementary figures and images for: Spectroscopic Characterization of Bovine, Avian and Johnin Purified Protein Derivative (PPD) with High-Throughput Fourier Transform InfraRed-Based Method
Source: Pathogens. 2019 Aug 29;8(3):136. doi: 10.3390/pathogens8030136 (PMC6789744; doi:10.3390/pathogens8030136)

Figure S1.

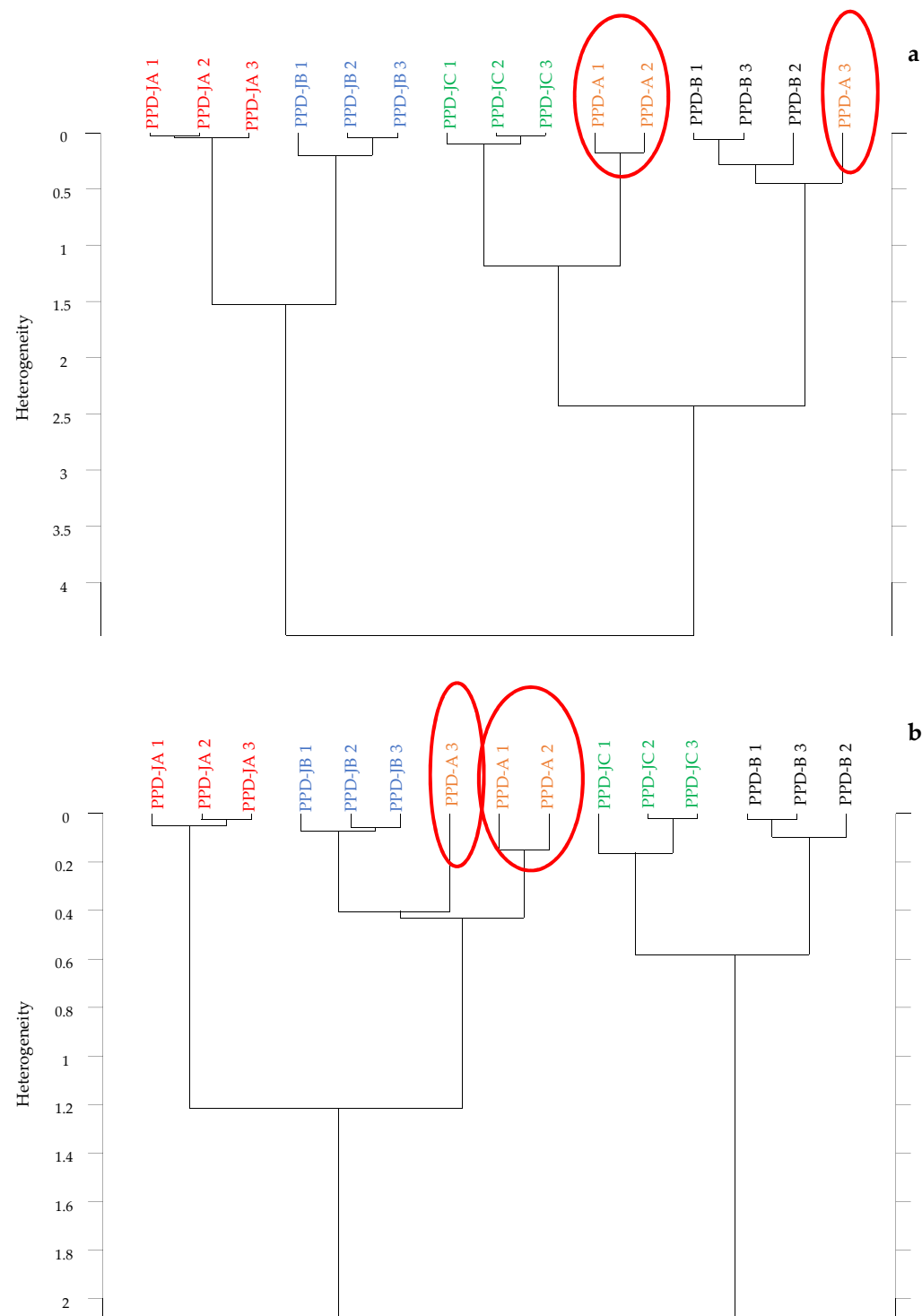

Figure S2.

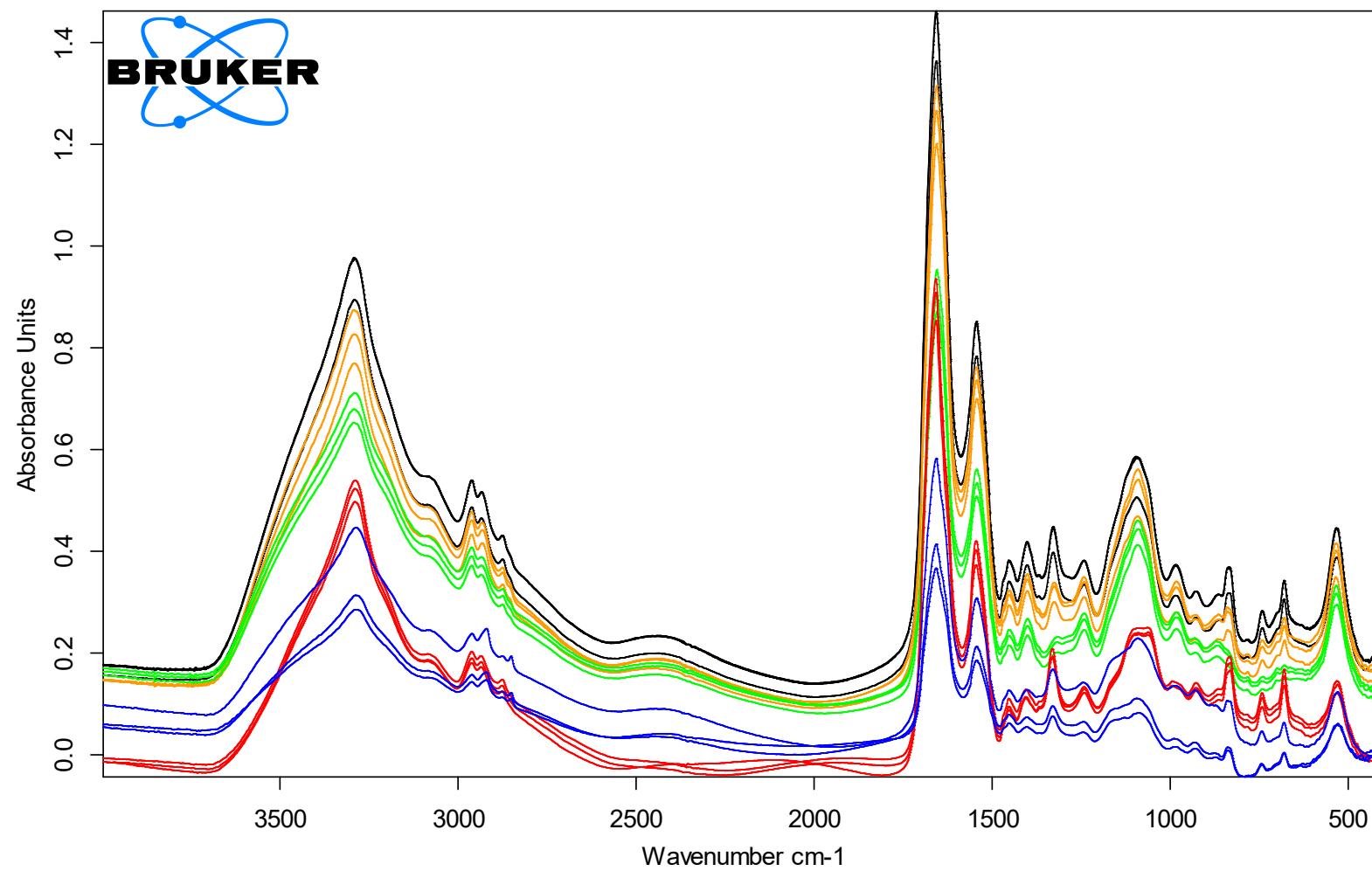

Supplement: Supplementary file 1 [file pathogens-08-00136-s001.pdf]
